# Rapid Clinical Improvement Temporally Associated with Off-Label LetiFend^®^ Vaccination in Canine Leishmaniasis: A Case Report

**DOI:** 10.3390/vaccines14090818

**Published:** 2026-09-17

**Authors:** Tobias Werner, Marius Rămneanţu, Torsten J. Naucke

**Affiliations:** 1 Institute of Analytical Chemistry, Mannheim University of Applied Sciences, Paul-Wittsack-Straße 10, 68163 Mannheim, Germany; 2 Parasitus Ex e.V., Research and Diagnostic Center for Vector-Borne Diseases, Deutzer Straße 64A, 53859 Niederkassel, Germany; tjnaucke@aol.com; 3 Small Animal Veterinary Practice Dr. Marius Ramneantu, Am Kufholz 10, 93138 Lappersdorf, Germany

**Keywords:** canine leishmaniasis, *Leishmania infantum*, veterinary vaccine, therapeutic vaccination, immunomodulation, LetiFend^®^

## Abstract

Canine leishmaniasis (CanL), caused by *Leishmania infantum*, can be difficult to manage in dogs with severe clinical disease, particularly when established treatments are impractical or poorly tolerated. We report an approximately 3-year-old mixed-breed dog from Sardinia with clinically manifest CanL, anemia, thrombocytopenia, marked hyperglobulinemia, and an immunofluorescence antibody test titer of 1:4000. The dog’s condition deteriorated during allopurinol treatment, with profound lethargy, vomiting, diarrhea, and complete refusal of food and water. Because escalation to combination leishmanicidal therapy (meglumine antimoniate or miltefosine) was considered clinically unsuitable in the severely debilitated patient, allopurinol monotherapy was discontinued after 18 days without clinical improvement, and one full commercial dose of the recombinant vaccine LetiFend^®^ was administered subcutaneously off-label. No immediate adverse reaction was observed. Clinical improvement became apparent within approximately 10 days, and near-normal activity returned within three weeks. During follow-up, hematological parameters normalized and serum protein electrophoresis showed an increased albumin/globulin ratio and a reduced gamma-globulin fraction. Domperidone was subsequently introduced as long-term immunomodulatory follow-up, and allopurinol was temporarily reintroduced after transient laboratory deterioration in 2024; the dog remained clinically healthy through July 2026. The close temporal association is clinically noteworthy but cannot establish causality. This hypothesis-generating observation supports controlled investigation of therapeutic vaccination as a potential complementary approach in canine leishmaniasis.

## 1. Introduction

Canine leishmaniasis (CanL), caused by *Leishmania infantum*, is a systemic vector-borne disease of major veterinary importance in endemic regions, including Southern Europe, South America, and parts of Asia [1,2]. The disease is transmitted by phlebotomine sand flies and may become life-threatening if untreated. Its complex pathophysiology, chronic progression, variable clinical manifestations, and high relapse rates complicate disease management.

According to the LeishVet consensus guidelines, treatment approaches are commonly differentiated between endemic and non-endemic areas [3]: endemic regions typically use leishmanicidal drugs (meglumine antimoniate, miltefosine), often combined with the leishmaniostatic agent allopurinol, though these carry risks of nephrotoxicity, hepatotoxicity, pancreatitis, and gastrointestinal disturbances [4,5,6,7] alongside rising drug-resistance concerns [8,9,10]. Non-endemic settings often favor leishmaniostatic approaches such as allopurinol, whose comparatively favorable toxicity profile is nonetheless tempered by risks of xanthinuria, xanthine urolithiasis [11,12], and resistance selection [13,14]. Alternative agents such as artesunate and α-bisabolol show antileishmanial activity, though evidence for routine use remains limited [15,16].

Immunomodulatory approaches aim to enhance or redirect the host immune response. Domperidone (Leisguard^®^ Ecuphar Veterinaria S.L.U., Sant Cugat del Vallès, Barcelona, Spain) has been associated with stimulation of cell-mediated immunity and improved outcomes in selected infected dogs [17,18,19,20]. Nutraceutical immunomodulators have also been investigated as adjuncts to antiparasitic therapy [21,22]. Vaccination, traditionally used for prevention, is increasingly being explored for therapeutic potential in CanL [23,24,25,26]. Reviews have summarized commercially available and recombinant vaccine strategies for canine leishmaniasis [27,28]. LetiFend^®^ (Laboratorios LETI S.L., Tres Cantos, Madrid, Spain) is a recombinant protein-based vaccine containing the chimeric Q protein [29,30,31] and demonstrated 72% efficacy in preventing clinical disease under field conditions [29]. It is authorized in the European Union for active immunization of non-infected dogs from six months of age [32]. The immunological rationale for vaccination against canine leishmaniasis has been described previously [33,34], and vaccine-based immunotherapy has produced clinical and immunological improvement in infected dogs in experimental and clinical studies [23,26].

We report a dog with severe CanL that showed rapid initial clinical improvement temporally associated with a single off-label administration of LetiFend^®^; the subsequent multi-year course, which included additional immunomodulatory and, transiently, leishmaniostatic treatment, is described separately. The clinical significance lies in the close temporal association between therapeutic vaccination and early recovery at a point when the dog was deteriorating and escalation to combination leishmanicidal therapy was considered clinically impractical. The report is descriptive and hypothesis-generating; it does not establish therapeutic efficacy.

## 2. Case Presentation

**Figure 1:**
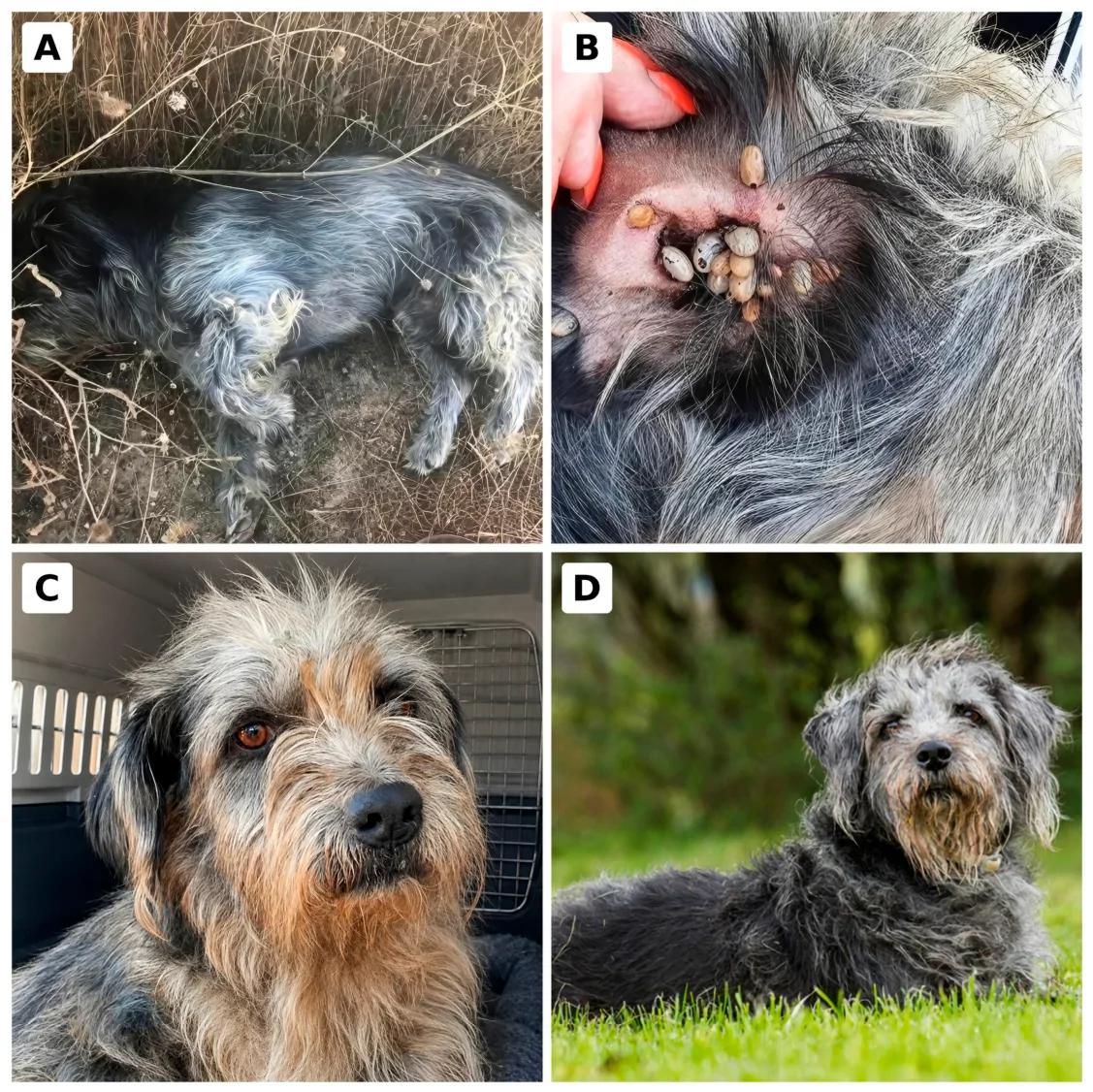
Clinical course from rescue to long-term recovery. (**A**) An approximately 3-year-old male mixed-breed dog found in the Sardinian outback in poor general condition. (**B**) The dog was heavily infested with at least 15 ticks identified as *Rhipicephalus sanguineus*. (**C**) Following antimicrobial treatment initiated because of the heavy tick infestation and suspected tick-borne infection, the dog gradually recovered and showed substantial clinical improvement. (**D**) Three weeks after therapeutic vaccination with LetiFend^®^, the dog had almost fully recovered and showed normal activity; he has remained clinically healthy for more than three years under domperidone-based follow-up, aside from a transient laboratory relapse in 2024 that resolved after temporary allopurinol reintroduction.

In summer 2021, a male mixed-breed dog, approximately three years old, was found in critical condition near a roadside in a remote part of Sardinia, Italy. The dog was markedly lethargic, severely dehydrated and anemic, and displayed extreme weakness and pale mucous membranes. Upon closer examination, the dog was found to be infested with at least 15 ticks identified as *Rhipicephalus sanguineus* (Figure 1). The heavy tick infestation raised suspicion of possible vector-borne infections, as *R. sanguineus* is a known vector of several canine hemopathogens.

Upon arrival at an animal shelter in Olbia, Sardinia, the dog received supportive care, including fluid therapy, hematinic supplementation, and antimicrobial treatment with doxycycline at 10 mg/kg PO SID for 28 days. The decision to administer doxycycline was based on the heavy tick infestation and its activity against *Ehrlichia* spp., *Anaplasma* spp., and other susceptible bacterial pathogens associated with *R. sanguineus*.

**Figure 2:**
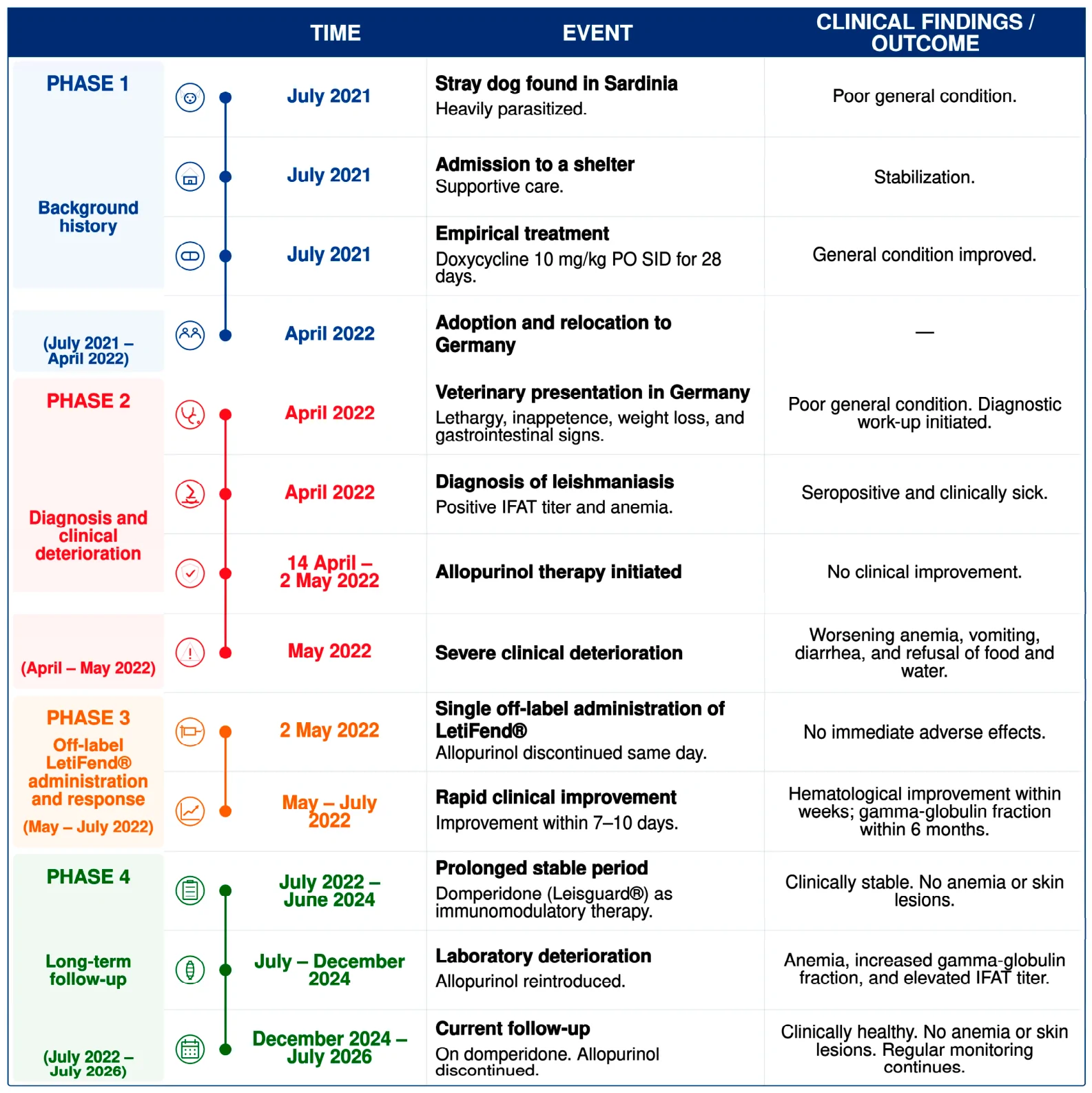
Clinical timeline of a dog with severe canine leishmaniasis. Timeline of a dog with severe clinically manifest canine leishmaniasis showing initial rescue in Sardinia, relocation to Germany, lack of clinical improvement during allopurinol therapy, discontinuation of allopurinol at the time of off-label LetiFend^®^ administration, rapid clinical improvement, and long-term clinical stability under immunomodulatory follow-up.

Over the following weeks, the dog gradually recovered, showing substantial clinical improvement. The dog regained body weight and physical strength and was assessed by shelter veterinary staff as clinically fit for transport; however, no reliable hematological data from this period in Sardinia were available for review, and the extent of laboratory normalization at that time cannot be confirmed. However, because systematic screening for vector-borne infections had not been performed at the initial presentation, chronic or subclinical infections, including leishmaniasis and other tick-borne infections, could not be excluded. In April 2022, an adoptive family was found, and the dog was relocated to Germany. Given its origin from an area endemic for *L. infantum* and its previous clinical history, the dog underwent a comprehensive veterinary examination and laboratory testing for infectious diseases commonly associated with the Mediterranean region. The complete clinical course, from initial rescue to long-term follow-up, is summarized in Figure 2.

### 2.1. Diagnostic Assessment

Clinical laboratory evaluation included hematology (scil Vet abc Plus+, scil Animal Care Company, Viernheim, Germany), serum protein capillary electrophoresis (MINICAP FLEX-PIERCING, Sebia, Lisses, France; six fractions: albumin, α1-, α2-, β1-, β2-, and γ-globulins), serum biochemistry (cobas 8000, Roche Diagnostics, Mannheim, Germany; performed by LABOKLIN GmbH & Co. KG, Bad Kissingen, Germany; urea, creatinine, total protein, albumin, globulin), and urinalysis (UA test strips; VetLab UA analyzer, IDEXX Laboratories, Westbrook, ME, USA).

Anti-*L. infantum* antibody titers were determined by semi-quantitative IFAT (MegaFLUO^®^ LEISH Kit, MEGACOR Diagnostik GmbH, Hörbranz, Austria) using serial dilutions from 1:50 to 1:8000 against *L. infantum* promastigote-coated slides, with fluorescence microscopy and a cut-off dilution of 1:100 for seropositivity.

On 14 April 2022, the dog tested strongly positive for *L. infantum*, with an IFAT titer of 1:4000, whereas serological testing for *E. canis* and *Anaplasma* spp. yielded negative results. Hematological examination showed lymphopenia, anemia, and thrombocytopenia. Urinalysis revealed erythrocyturia (30 Ery/µL) and proteinuria (250 mg/L) by dipstick analysis. Serum protein electrophoresis revealed marked hyperglobulinemia, with an albumin/globulin ratio of 0.4 and a substantially increased gamma-globulin fraction (Table 1).

Given the previous heavy infestation with *R. sanguineus*, differential diagnoses included co-infections with other vector-borne pathogens, particularly *E. canis* and *Anaplasma* spp. Although serological testing for these pathogens was negative at the time of evaluation, the absence of systematic diagnostic screening at the time of rescue precludes complete exclusion of previous or transient co-infections. Other potential causes of anemia and hyperglobulinemia were considered less likely in view of the high anti-Leishmania antibody titer, the compatible clinical presentation, and the characteristic alterations observed by serum protein electrophoresis.

According to the LeishVet guidelines for the practical management of CanL [3], the dog was classified as seropositive and clinically sick, with findings consistent with clinically manifest CanL. This classification was supported by the high anti-*L. infantum* antibody titer (IFAT 1:4000), marked anemia and thrombocytopenia, hyperglobulinemia, a markedly decreased albumin/globulin ratio of 0.4, proteinuria detected by dipstick analysis, and severe deterioration of the general clinical condition.

However, formal assignment to a specific LeishVet clinical stage (I–IV) was not possible because the urinary protein-to-creatinine ratio (UPC) was unavailable. Since serum creatinine and urea concentrations remained within their respective reference ranges, advanced renal involvement could not be demonstrated on the basis of the available data.

### 2.2. Therapeutic Intervention

On the same day (14 April 2022), treatment with allopurinol at 5 mg/kg twice daily was initiated. No clinical improvement was observed, and the dog’s condition deteriorated markedly over the following weeks. The dog stopped eating and drinking voluntarily, developed diarrhea and vomiting, and showed signs of worsening anemia, including increasingly pale mucous membranes. It became profoundly apathetic and lethargic and eventually left its resting place only to vomit.

At this point, the dog was severely compromised despite ongoing allopurinol treatment. Oral miltefosine was considered impractical because active vomiting and complete refusal of food and water made reliable administration and absorption uncertain, while its potential gastrointestinal adverse effects were of particular concern. Meglumine antimoniate requires repeated, typically twice-daily, subcutaneous injections over several weeks and was considered separately unsuitable in this patient: adverse effects, including painful injection-site reactions, systemic reactions with lethargy and anorexia, and acute renal failure, have been reported in up to approximately 30% of treated dogs, with treatment discontinuation required in roughly 15% [35], and isolated fatal outcomes associated with treatment-related acute renal failure have been documented, including in dogs without pre-existing renal impairment [36,37]. Given the dog’s already severely compromised general condition, the risk of exacerbating systemic or renal compromise was judged to outweigh the expected benefit of escalating to meglumine antimoniate at this stage.

Because allopurinol had not been associated with clinical improvement and the dog’s condition continued to deteriorate, it was discontinued after 18 days of treatment, on 2 May 2022, at the time of vaccination. One full commercial dose of LetiFend^®^ was administered subcutaneously off-label, based on its labeled pharmacological rationale of stimulating a parasite-specific host immune response—a rationale not directly confirmed in this dog, as neither antigen-specific cellular responses nor parasite burden were measured. No conventional leishmanicidal drug was administered. This therapeutic use was undertaken under the cascade provisions of Regulation (EU) 2019/6 on veterinary medicinal products [38]. The decision was additionally supported by evidence that vaccine-based immunotherapy can be administered to infected dogs without necessarily exacerbating disease [39] and that LetiFend^®^ can exert immunological effects in experimentally infected dogs [40]. According to the vaccine’s marketing authorization, LetiFend^®^ is considered safe for use in already-infected dogs, and re-vaccination of infected animals has not been associated with a worsening of disease course during observation, although efficacy in this population has not been established [32].

No immediate adverse reaction attributable to vaccination was observed. At the time of vaccination, the dog could only be fed a watery mash prepared from its preferred food. Approximately 10 days after LetiFend^®^ administration, its general condition began to improve noticeably. Within three weeks, appetite, spontaneous water intake, behavior, and exercise tolerance had recovered, allowing unrestricted daily physical activity, including walks of more than 3 km.

### 2.3. Follow-Up and Outcomes

Following the rapid initial clinical recovery, the dog was regularly monitored to assess its clinical condition and laboratory parameters. The course of hematological, serological, urinary, and serum protein parameters over the 24-month follow-up period is summarized in Table 1. The rapid clinical improvement was followed by normalization of the major hematological abnormalities. As shown in Table 1, erythrocyte count, hemoglobin concentration, hematocrit, and platelet count had returned to their respective reference ranges at the six-month follow-up and remained stable thereafter.

**Figure 3:**
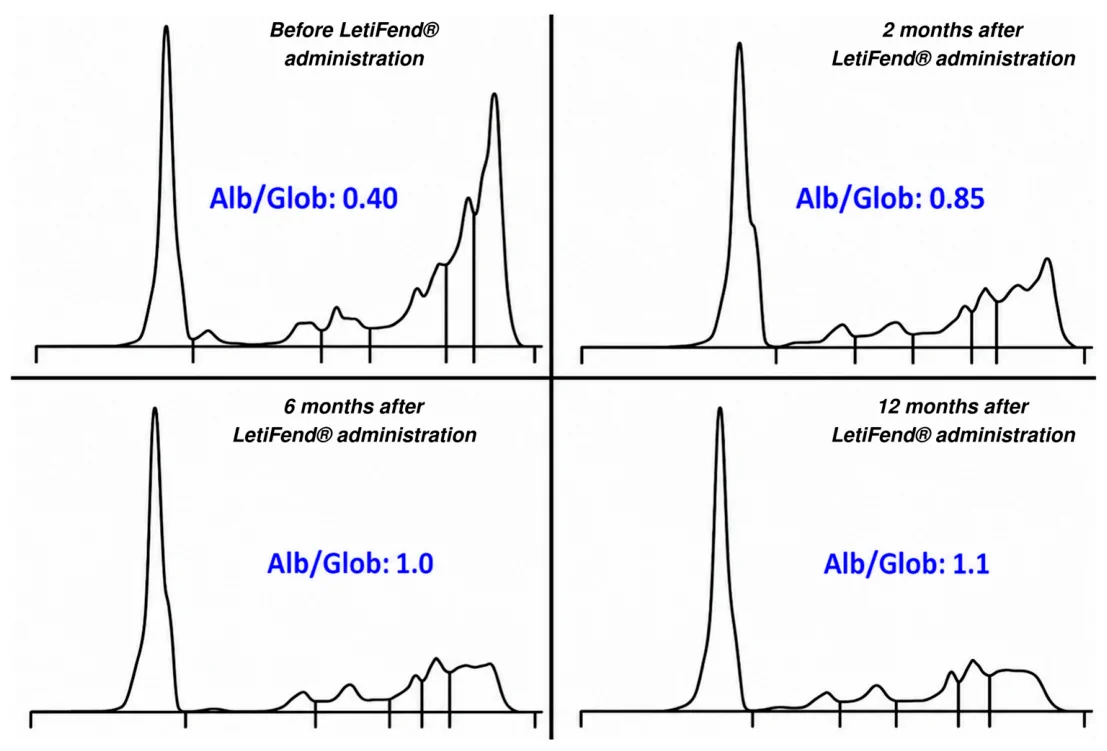
Serum protein capillary electrophoresis before and at 2, 6, and 12 months after LetiFend^®^ administration, showing a reduction in the gamma-globulin fraction and improvement in the albumin/globulin ratio. (Alb/Glob, the albumin-to-globulin ratio, is calculated by dividing the serum albumin concentration by the combined concentration of all globulin fractions.) The 2-month timepoint was recorded in addition to the standard 0/6/12/24-month monitoring schedule reported in Table 1. The electropherogram at 24 months was not retained as an image file; the corresponding numerical values for this timepoint are reported in Table 1 only. The original electropherograms are provided in Figure S1 of the Supplementary Materials.

Marked changes were also observed in serum protein electrophoresis. The albumin/globulin ratio increased from 0.4 at baseline to 1.0 after six months and remained within the normal range throughout the subsequent observation period. Serum protein capillary electrophoresis demonstrated a pronounced reduction in the gamma-globulin fraction during the same period (Figure 3).

Despite the marked clinical and hematological improvement, the IFAT antibody titers did not show a sustained decline. The titer initially decreased from 1:4000 at baseline to 1:1000 after six months, subsequently increased to 1:2000 after 12 months, and returned to 1:4000 after 24 months. This dissociation emphasizes that serological titers alone do not necessarily parallel clinical disease activity or effective control of infection.

During the first two years following LetiFend^®^ administration, the dog received domperidone at four-month intervals, while no further leishmaniostatic or leishmanicidal treatment was administered. Between July and December 2024, recurrence of anemia together with an increased gamma-globulin fraction and elevated IFAT titer led to reintroduction of allopurinol according to a staged protocol used in non-endemic areas [41]. Allopurinol was subsequently discontinued after stabilization. At the latest follow-up in July 2026, the dog remained clinically healthy without anemia or skin lesions while receiving domperidone and regular clinical monitoring.

## 3. Discussion

The clinical history of this dog illustrates a common diagnostic problem in rescued dogs from endemic regions: infections may remain undetected when systematic screening is not performed at the time of rescue. The absence of systematic screening at the time of rescue resulted in an initially undiagnosed *L. infantum* infection and limited the ability to fully reconstruct the early disease course. Comprehensive diagnostic screening should therefore be considered in dogs from endemic areas, even when they initially recover following supportive or empirical antimicrobial treatment.

At diagnosis, the high IFAT antibody titer together with the clinical, hematological, and electrophoretic abnormalities indicated substantial disease activity. According to the LeishVet guidelines, the dog was classified as seropositive and clinically sick [3]. However, the absence of a UPC precluded definitive assignment to a specific LeishVet clinical stage. The dog began to recover rapidly after a single administration of LetiFend^®^ on 2 May 2022, despite having deteriorated during 18 days of preceding allopurinol monotherapy (14 April–2 May 2022). Recovery subsequently occurred without any further leishmanicidal or leishmaniostatic drug administered at that time, although a delayed contribution of the immediately preceding allopurinol course cannot be entirely excluded and is examined further below.

To further evaluate whether the improvement could instead reflect a delayed effect of the preceding allopurinol course, additional data—now included in Table 1 as an interim timepoint (T2)—are informative. Hematology and serum protein electrophoresis performed approximately 7–9.5 weeks after vaccination (22–23 June 2022), together with clinical chemistry performed two weeks later (7 July 2022), all before domperidone was introduced on 31 July 2022, showed near-complete normalization of erythrocyte and platelet parameters (hemoglobin 14.5 g/dL, hematocrit 44.0%, platelets 519 × 10^3^/µL) and an improved albumin/globulin ratio (0.85 by electrophoresis; 1.1 by clinical chemistry two weeks later), whereas the gamma-globulin fraction (25.9% of total protein) and IFAT titer (1:4000, unchanged from baseline) had not yet substantially improved. This pattern—early hematological recovery preceding a slower decline in gamma-globulins and antibody titer—is consistent with the expected kinetics of resolving antigen-driven inflammation and argues against a delayed carry-over effect of allopurinol, since no clinical or hematological improvement had occurred during the preceding 18 days of allopurinol administration itself. In addition, the reported adverse-effect profile of allopurinol in dogs is predominantly urinary (xanthinuria, xanthine urolithiasis), with a median reported time to onset of 150 days [42]; gastrointestinal signs are not an established adverse effect at the therapeutic dose used here (5 mg/kg twice daily), and the concurrent progressive anemia observed during allopurinol treatment is not readily explained by a drug-related mechanism, since allopurinol is not reported to cause myelosuppression or hemolysis in dogs. We therefore regard progression of CanL, rather than an adverse drug reaction to or a delayed effect of allopurinol, as the more likely explanation for the pre-vaccination deterioration and subsequent recovery, although neither alternative can be formally excluded given the retrospective, single-case design of this report.

From an immunopathological perspective, effective control of *L. infantum* infection is associated with cell-mediated immunity and macrophage-mediated intracellular parasite killing, whereas progressive disease is frequently accompanied by pronounced humoral responses, hypergammaglobulinemia, and pathogenic immune-complex formation.

In this context, the marked increase in the albumin/globulin ratio and the reduction in the gamma-globulin fraction after clinical recovery may be clinically relevant. IgG-containing immune complexes contribute to the immunopathogenesis and progression of leishmaniasis [43], and experimental data indicate that LetiFend^®^ vaccination can reduce circulating immune complexes in dogs experimentally infected with *L. infantum* [40]. The electrophoretic changes observed in this dog are therefore consistent with an altered humoral immune profile, although a direct causal relationship with vaccination cannot be established.

However, these findings do not provide conclusive evidence of a vaccine-induced shift toward a Th1-dominated immune response, as neither antigen-specific T-cell responses nor relevant cytokines were measured. The proposed immunological mechanism therefore remains hypothetical, although it is supported by the temporal clinical course, changes in serum protein profiles, and current understanding of CanL immunobiology.

The recombinant Q protein contained in LetiFend^®^ is designed to stimulate cell-mediated immune responses against Leishmania antigens. Vaccination might therefore enhance or redirect an existing parasite-specific immune response in some infected dogs; whether this occurred in the present case remains unconfirmed, as neither parasite burden nor antigen-specific cellular responses were measured. Previous therapeutic vaccination studies have reported improvements in clinical and immune status and reductions in parasite burden [23], although additional immunotherapy may be required for sustained parasite control [26]. Direct clinical evidence for therapeutic use of LetiFend^®^ in dogs with severe naturally acquired CanL, however, remains limited.

Several alternative explanations for the observed improvement need to be considered. Spontaneous clinical improvement cannot be excluded. The close temporal relationship to vaccination and the preceding severe deterioration are noteworthy but do not reduce this uncertainty sufficiently to support a causal inference. Similarly, a delayed contribution from the preceding allopurinol treatment cannot be completely ruled out. However, no clinical improvement was observed during its administration, and recovery occurred after its discontinuation. Prior doxycycline treatment is unlikely to explain the rapid improvement because it had been administered many months earlier. Previous or transient co-infections nevertheless cannot be completely excluded because comprehensive screening was not performed at the time of rescue.

Supportive care, improved husbandry, and nutritional support may also have contributed to stabilization. However, immediately before vaccination, the dog was severely compromised and refused both food and water. The subsequent restoration of appetite, spontaneous water intake, activity, and exercise tolerance therefore represented a clinically meaningful change in the disease course.

Clinical recovery was not accompanied by a sustained decline in IFAT antibody titers. Despite marked clinical and hematological improvement, the antibody titer did not show a sustained decline and eventually returned to its initial level. This illustrates the limitations of using antibody titers alone as markers of clinical disease activity. Our assessment of clinical improvement therefore relied primarily on hematological normalization and serum protein electrophoretic changes rather than on antibody titers.

Beginning in July 2022, the dog received domperidone at four-month intervals, and allopurinol was temporarily reintroduced after laboratory deterioration in 2024. Both interventions may have contributed to long-term clinical stability. Therefore, only the rapid initial recovery showed a close temporal relationship with vaccination; the prolonged course cannot be attributed to vaccination alone. As a single-case observation without a control group, conclusions regarding causality remain limited. Diagnostic evaluation was incomplete at initial rescue; parasite burden (e.g., in blood, bone marrow, lymph nodes, or skin) and cellular immune responses were not directly assessed, and the absence of a UPC value prevented definitive LeishVet staging. Domperidone administration during long-term follow-up represents an additional potential confounding factor. This absence of invasive parasitological and immunological data reflects the nature of the case: a privately owned dog managed in general veterinary practice rather than within a research protocol. Bone marrow or lymph node aspiration was not clinically indicated and could not be justified without a dedicated research consent framework, and no material was collected for parasite isolation, culture, or subsequent molecular identification and genetic characterization. Even a less invasive parasitological approach would not necessarily have resolved this limitation, as PCR sensitivity varies substantially according to the biological specimen and molecular assay used: TaqMan-qPCR detection rates in experimentally infected dogs ranged from 13% in peripheral blood to 73% in conjunctival swabs, 76% in bone marrow aspirates, and 86% in popliteal lymph node aspirates [44], while in naturally infected, symptomatic dogs, latent-class-corrected conjunctival swab sensitivity was only 29.2–37.5% [45]. A negative qPCR result from a single specimen, including a minimally invasive conjunctival swab, would therefore not have reliably excluded infection or quantified parasite burden in this case. Moreover, invasive tissue sampling of this kind is not part of the routine clinical management of Leishmania-infected dogs, for whom treatment decisions are guided by clinical, serological, and biochemical staging rather than tissue-level parasite quantification [3].

Even with these limitations, the temporal association between LetiFend^®^ administration and rapid clinical recovery deserves attention, particularly because the improvement was followed by normalization of hematological parameters and marked electrophoretic changes. Therapeutic vaccination should not be considered a replacement for established treatment protocols. The present observation instead supports controlled prospective investigation of its safety and potential complementary role, particularly when established therapy is impractical or poorly tolerated.

## 4. Conclusions

This case describes rapid clinical improvement, temporally associated with a single off-label subcutaneous administration of LetiFend^®^, in a dog with severe clinically manifest CanL that had shown no improvement during 18 days of preceding allopurinol monotherapy. Recovery became apparent within approximately 10 days after vaccination, with near-normal activity restored within three weeks. Hematological parameters subsequently normalized, and serum protein electrophoresis showed a marked reduction in the gamma-globulin fraction and improvement in the albumin/globulin ratio.

The dog remained clinically healthy at the latest follow-up in July 2026. Domperidone was administered during long-term follow-up, and allopurinol was temporarily reintroduced after laboratory deterioration in 2024; the prolonged clinical course therefore cannot be attributed to vaccination alone. The close temporal association between vaccination and initial recovery is noteworthy but cannot establish causality. This case should be regarded as a hypothesis-generating clinical observation supporting controlled investigation of therapeutic vaccination as a potential complementary approach in canine leishmaniasis.

## Figures and Tables

**Table 1:** Laboratory parameters recorded at baseline (T0) and 6 (T6), 12 (T12), and 24 (T24) months after diagnosis. * All measured serum creatinine and urea concentrations remained within their respective reference ranges throughout the observation period. T2 denotes an interim assessment performed approximately 7–9.5 weeks after vaccination (22 June–7 July 2022), before domperidone was introduced on 31 July 2022; values combining electrophoresis percentages with total protein measured two weeks apart are marked in the Discussion, where directly measured clinical-chemistry values from the same period are also reported. Urinalysis was not repeated at this timepoint (n/a).

Substrate	Parameter	T0	T2	T6	T12	T24	Reference Range
Serum *	Total protein (g/L)	83.6	81.5	67.2	67.9	79.0	54.0–75.0
	Alb/Glob ratio	0.4	0.85	1.0	1.1	1.0	>0.6
	Gamma globulin (g/L)	24.1	21.1	12.4	12.7	16.5	
	Beta 2 globulin (g/L)	13.0	6.7	6.7	6.7	8.7	
	Beta 1 globulin (g/L)	13.1	7.1	4.2	4.7	7.7	
	Alpha 2 globulin (g/L)	4.9	5.2	4.2	5.2	5.3	
	Alpha 1 globulin (g/L)	4.8	3.9	3.4	3.8	3.7	
	Total globulins (g/L)	59.9	44.0	30.9	33.1	41.9	<45
	Albumin (g/L)	23.8	37.5	34.4	34.8	37.9	25.0–44.0
	Anti-*Leishmania* IFAT titer	1:4000	1:4000	1:1000	1:2000	1:4000	Negative
Blood	WBC (10^3^/µL)	4.3	8.3	9.3	8.1	6.6	6.0–12.0
	RBC (10^6^/µL)	3.9	6.3	5.7	6.0	6.3	5.5–8.5
	Hb (g/dL)	8.9	14.5	15.4	15.6	15.2	15.0–20.0
	Hct (%)	25.1	44.0	44.1	44.6	45.7	44.0–57.0
	PLT (K/µL)	98.0	519	237	202	229	200–460
Urine	Ery (Ery/µL)	30	n/a	Negative	Negative	Negative	Negative
	Protein (mg/L)	250	n/a	Negative	Negative	Negative	Negative

## Data Availability

All data supporting the findings of this case report are contained within the article. Further inquiries can be directed to the corresponding author.

## Supplementary Materials

The following supporting information can be downloaded at: https://www.mdpi.com/article/10.3390/vaccines14090818/s1, Figure S1: Original serum protein capillary electropherogram tracings for the dog at baseline (T0) and 2 (T2), 6 (T6), and 12 (T12) months after vaccination, corresponding to the summarized data shown in Figure 3 and Table 1.

## Abbreviations

The following abbreviations are used in this manuscript:

Alb	Albumin
CanL	Canine leishmaniasis
EDTA	Ethylenediaminetetraacetic acid
Ery	Erythrocytes
FITC	Fluorescein isothiocyanate
Glob	Globulin
Hb	Hemoglobin
Hct	Hematocrit
IFAT	Immunofluorescence antibody test
IgG	Immunoglobulin G
NK	Natural killer
PLT	Platelet count
RBC	Red blood cell count
Th1	T helper 1 cells
UPC	Urinary protein-to-creatinine ratio
WBC	White blood cell count
