# Supplementary material for: Rapid Clinical Improvement Temporally Associated with Off-Label LetiFend® Vaccination in Canine Leishmaniasis: A Case Report
Source: Vaccines (Basel). 2026 Sep 17;14(9):818. doi: 10.3390/vaccines14090818 (PMC13611871; doi:10.3390/vaccines14090818)
Supplement: Supplementary file 1 [file vaccines-14-00818-s001.zip › vaccines-4536561-supplementary.pdf]

Figure S1: Original serum protein capillary electropherogram tracings for the dog at baseline (T0) and 2 (T2), 6 (T6), and 12 (T12) months after vaccination

Parasitus Ex e.V.

Verein zur Förderung der Grundlagenforschung von Infektionskrankheiten bei Tieren

Patientenname: **Hd Lorenzo** Untersuchung vom: **20/04/2022**

Besitzer: **Sardinienhunde** Probe Nr.: **26**

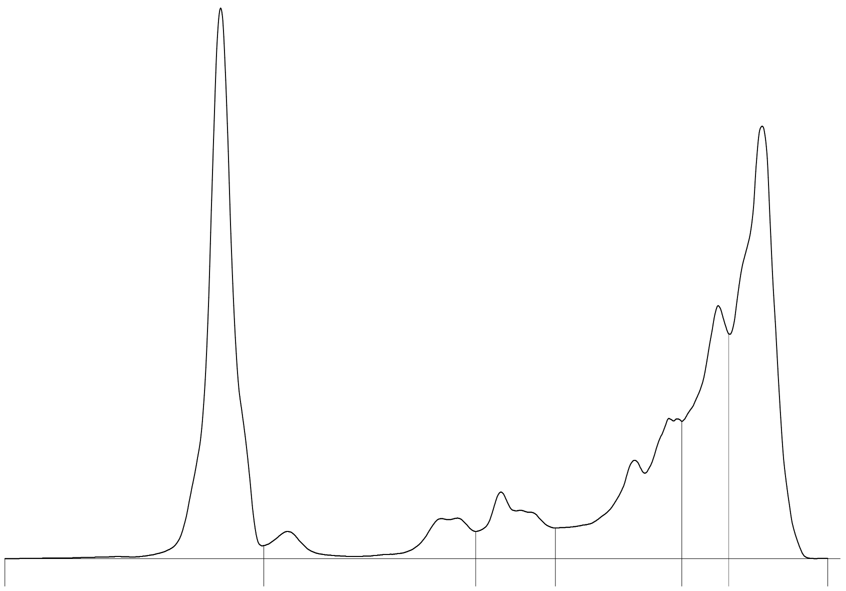

Serumprotein Kapillar-Elektrophorese

Fraktion	%	g/L
Albumin	28,5	23,8
Alpha 1	5,5	4,6
Alpha 2	5,9	4,9
Beta 1	15,7	13,1
Beta 2	15,6	13,0
Gamma	28,8	24,1

Alb/Glob: **0,40** **Gesamteiweiß:** **83,6 g/l**

Kommentare:

Parasitus Ex e.V.

Verein zur Förderung der Grundlagenforschung von Infektionskrankheiten bei Tieren

Patientenname:	**Hd Lorenzo**	Untersuchung vom:	**23/06/2022**
Besitzer:	**Sardinienhunde**	Probe Nr.:	**21**

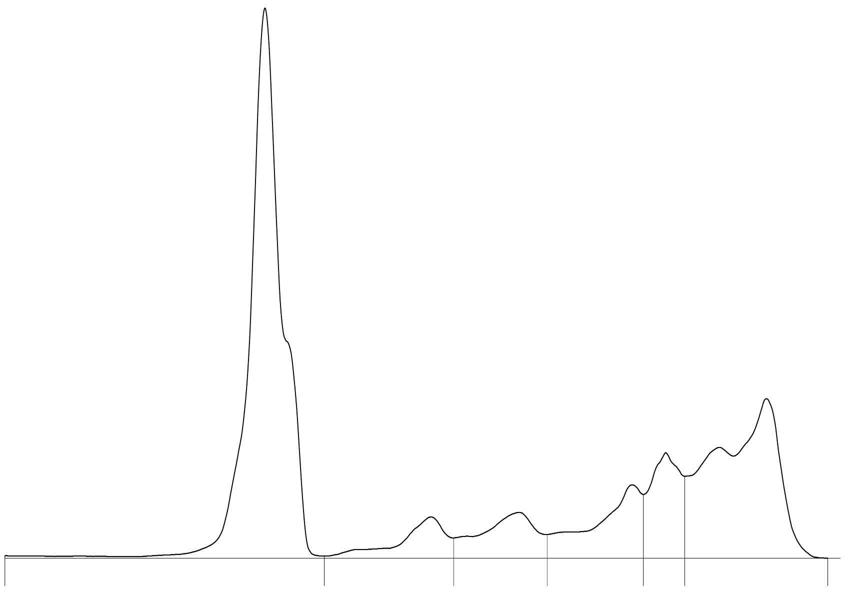

Serumprotein Kapillar-Elektrophorese

Fraktion	%
Albumin	**46,0**
Alpha 1	**4,8**
Alpha 2	**6,4**
Beta 1	**8,7**
Beta 2	**8,2**
Gamma	**25,9**

Alb/Glob: **0,85** **Gesamteiweiß:** **g/l**

Kommentare:

Parasitus Ex e.V.

Verein zur Förderung der Grundlagenforschung von Infektionskrankheiten bei Tieren

Patientenname: **Hd Lorenzo**

Untersuchung vom: **14/10/2022**

Besitzer: **Sardinienhunde**

Probe Nr.: **29**

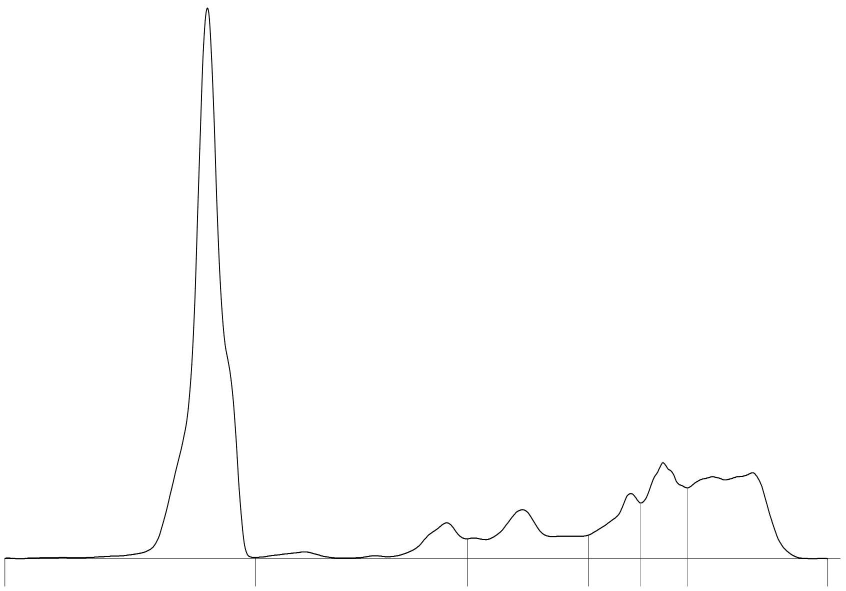

Serumprotein Kapillar-Elektrophorese

Fraktion	%	g/L
Albumin	51,1	34,3
Alpha 1	5,0	3,4
Alpha 2	9,2	6,2
Beta 1	6,3	4,2
Beta 2	10,0	6,7
Gamma	18,4	12,4

Alb/Glob: **1,04**

Gesamteiweiß: **67,2 g/l**

Kommentare:

Patientenname: **Hd Lorenzo**

Untersuchung vom: **26/04/2023**

Besitzer: **Sardinienhunde**

Probe Nr.: **33**

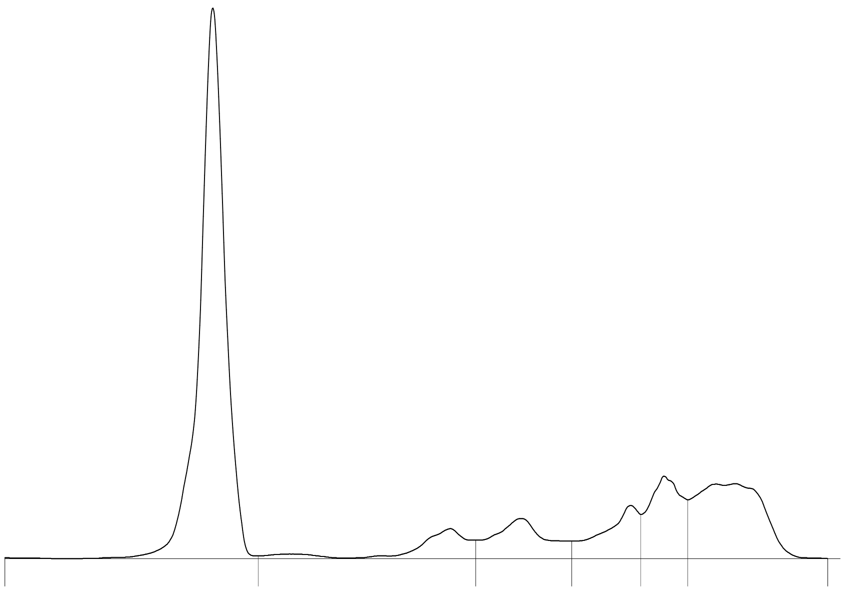

Serumprotein Kapillar-Elektrophorese

Fraktion	%	g/L
Albumin	**51,3**	**34,8**
Alpha 1	**5,6**	**3,8**
Alpha 2	**7,7**	**5,2**
Beta 1	**6,9**	**4,7**
Beta 2	**9,8**	**6,7**
Gamma	**18,7**	**12,7**

Alb/Glob: **1,05**

Gesamteiweiß: **67,9** **g/l**

Kommentare:
